# When competence isn’t enough: age and gender stereotypes in recruitment practices in Czechia

**DOI:** 10.3389/fpsyg.2026.1795235

**Published:** 2026-05-01

**Authors:** Martina Rašticová, Štěpán Konečný, Jakub Šácha, Martin Lakomý, Arpit Tripathi, Pawan Kumar Mishra

**Affiliations:** 1 Faculty of Business and Economics, Mendel University in Brno, Brno, Czechia; 2 Brno University of Technology, Brno, Czechia

**Keywords:** age bias, candidate evaluation, competency-based hiring, digitalization, gender bias, HR decision-making, recruitment

## Abstract

This study explores the intersection of age and gender biases in recruitment, particularly in the context of digitalization, where evolving job demands are reshaping the hiring landscape. Using an experimental design, we examine how HR managers in the Czech Republic assess candidates with similar qualifications but varying in age (32 and 56 years) and gender (male and female) for digitally demanding roles. A representative sample of 608 HR professionals evaluated candidate profiles, allowing us to isolate the impact of demographic factors on perceptions of key competencies such as digital proficiency, interpersonal skills, and adaptability. Our findings show that age and gender influence candidate evaluations in early recruitment, even when qualifications are equivalent. Although explicit recommendation scores were not statistically different across profiles, younger candidates, especially younger women, tended to receive more favorable impressions than older male candidates. Older applicants were often viewed as technically strong and creative, but younger applicants were more frequently associated with adaptability and interpersonal ease, which are increasingly emphasized in digital work settings. These perception patterns reflect demographic expectations rather than verified competencies and suggest that hiring decisions can be shaped by assumptions linked to age and gender rather than by the documented strengths of the applicant. In light of labor market shortages in the European Union, this research emphasizes the importance of inclusive recruitment strategies. This study contributes a novel experimental approach in Central Europe, particularly within the Czech context, where research on these biases remains scarce, and offers directions for future inquiry into the effects of digitalization on recruitment practices.

## Introduction

Digital transformation and demographic aging are reshaping European labor markets, yet their combined effects on recruitment remain uneven. In 2023, only 56% of EU adults aged 16–74 possessed at least basic digital skills, with substantially lower rates among individuals aged 55–74 (Eurostat, 2023). At the same time, employers across the European Union report persistent difficulty filling digitally intensive roles, reflecting a widening digital skills gap (European Commission, 2023). These trends coincide with ongoing demographic disparities in hiring: recent audit and experimental evidence continues to document lower callback rates for older applicants and persistent gender segregation in technology-oriented fields (Neumark et al., 2019; Lössbroek et al., 2021; Young et al., 2023). In the Czech Republic, workforce aging and digital skill inequalities mirror broader EU patterns, yet experimental evidence on how HR professionals evaluate age and gender in digitally demanding roles remains limited. Together, these conditions create a measurable and timely problem: as digital competence becomes central to employability, demographic stereotypes may shape recruitment decisions in ways that are not yet fully understood.

Recruitment practices are increasingly shaped by digital transformation (Trauth et al., 2016; Handel, 2016; Hirsch-Kreinsen, 2016). Organizations now rely on digitally mediated screening tools and prioritize roles in which digital competence is treated as a central indicator of employability. Digitalization is often presented as a mechanism that improves efficiency and standardizes evaluation, with the potential to reduce human bias (Tiru and Mohorâta, 2020; Hullman et al., 2010; Bernhardt et al., 2002). However, emerging evidence suggests that digital systems do not automatically eliminate inequality. Instead, they may alter the way bias is expressed, shifting attention toward competence-related inferences linked to adaptability, learning speed, and technological fluency (Segkouli et al., 2023).

Two stereotype systems are particularly relevant in this context (Dovidio et al., 2010; Abrams et al., 2016). Ageism, defined as stereotyping and discrimination based on age, continues to affect older workers’ access to employment and advancement (Cebola et al., 2023; Turek and Henkens, 2020; Schreurs et al., 2017; Zacher et al., 2021; van Selm and Heijkant, 2021). Older applicants are often perceived as less adaptable to technological change despite evidence that they can successfully acquire digital skills when training is available (Kleissner and Jahn, 2020). Gender stereotypes also influence competence evaluations, especially in technical and digitally intensive domains where expertise is culturally associated with men (Wynn and Correll, 2017; Young et al., 2023; Heilman and Parks-Stamm, 2007; Williams and Polman, 2015; Kenny et al., 2016). When age and gender cues intersect, patterns of evaluation may become more complex (Bokek-Cohen, 2018; Almukhambetova et al., 2021; Dudová and Pospíšilová, 2022). Research on gendered ageism suggests compounded disadvantage for older women (Mariano et al., 2020), yet digital competence norms may also produce differentiated patterns depending on which traits are emphasized in evaluation.

This study examines a specific theoretical tension regarding bias under digitalization. Digital transformation may intensify demographic disadvantage in hiring, but it may also restructure bias toward competence- and vitality-linked dimensions associated with contemporary “ideal worker” expectations. In such a case, explicit hiring recommendations may appear neutral, while trait-level impressions and perceived skill gaps still reflect systematic demographic differences. To test this proposition, we use a 2 × 2 between-subject factorial vignette design in which applicant qualifications are held constant and only age and gender cues vary. This design allows us to assess whether demographic cues influence (a) hiring recommendation, (b) perceived skill gaps in digital and related domains, and (c) multidimensional trait impressions.

### Age, gender, and competence stereotypes in digital work contexts

Age-related stereotypes are particularly salient in digitally transforming labor markets because employability is increasingly linked to perceived adaptability and technological competence. Older workers are frequently described as less flexible, slower to learn new systems, or less comfortable with digital tools (Burnes et al., 2019; Bellotti et al., 2023). These perceptions persist despite evidence that older employees can successfully develop digital skills when training and institutional support are available (Kleissner and Jahn, 2020). Recruitment decisions are often made under time constraints and rely on heuristic judgments. When digital competence is implicitly associated with youth, older applicants may be evaluated as less adaptable regardless of their qualifications. Such evaluations can shape trait-level judgments concerning vitality, flexibility, and problem-solving ability, which are central to digitally demanding roles.

Gender stereotypes operate through a comparable mechanism. Technical and digital competence is frequently coded as masculine, whereas women are more often associated with communal or relational attributes (Diekman and Eagly, 2000; Wynn and Correll, 2017). Even when women demonstrate equivalent performance in technical domains, they may be perceived as less competent or less suitable for leadership in digitally intensive positions (Cundiff et al., 2018). These stereotypes influence both career pathways and hiring evaluations by shaping expectations about who fits digital work environments. Digital transformation may therefore not eliminate gender bias but redirect it toward assessments of technical credibility and perceived expertise.

Intersectionality introduces additional complexity. Age and gender cues may interact rather than operate independently. Research on gendered ageism indicates that older women can experience compounded disadvantage in employment settings (Handy and Davy, 2007). However, intersectional effects are not necessarily uniform across all evaluative dimensions. In contexts where digital competence is strongly associated with youth, younger women may receive relatively favorable evaluations, whereas older men may face penalties concentrated in competence-linked domains. These competing possibilities generate a clear theoretical question: does digitalization intensify cumulative disadvantage, or does it restructure bias by altering which stereotype dimensions become most salient during evaluation?

### Problem statement

Digital transformation has increased employer demand for technological competence and adaptability, yet demographic disparities in hiring persist. Recent field and audit studies demonstrate that older applicants continue to experience lower callback rates compared to younger applicants, even when qualifications are equivalent (Neumark et al., 2019; Lössbroek et al., 2021). At the same time, women remain underrepresented in digitally intensive and technical roles, despite comparable educational attainment in many labor markets (Young et al., 2023). These patterns indicate that structural inequalities remain embedded in recruitment processes (Acker, 2000; Rhode, 2012; Jyrkinen and McKie, 2012).

As organizations increasingly prioritize digital skills, evaluative emphasis may shift toward perceived adaptability, learning speed, and technological fluency. These attributes are often stereotypically associated with youth and masculinity. Empirical evidence suggests that older workers are frequently perceived as less technologically adaptable despite documented capacity for digital skill acquisition (Kleissner and Jahn, 2020; Drazic and Schermuly, 2024). However, it remains unclear whether digital transformation intensifies demographic disadvantage, restructures bias toward competence-related dimensions, or produces differentiated intersectional effects.

The absence of controlled experimental studies that isolate age and gender cues while holding qualifications constant limits our understanding of how bias operates in digitally demanding recruitment contexts. Without such evidence, organizations lack clear guidance on whether digital hiring systems reduce, reproduce, or reconfigure demographic inequality. This unresolved issue represents a measurable and actionable research problem that warrants systematic investigation.

### Knowledge gap and theoretical contribution

Age and gender stereotypes remain well documented in employment research. Recent audit and experimental studies confirm that older applicants continue to experience disadvantages in hiring, even when qualifications are comparable (Neumark et al., 2019; Lössbroek et al., 2021). Similarly, gender-based competence biases persist in technical and leadership domains, shaping how candidates are evaluated for roles requiring authority and expertise (Young et al., 2023; Cundiff et al., 2018). Although digital transformation has altered recruitment processes, evidence suggests that technological systems do not automatically eliminate these biases (Tiru and Mohorâta, 2020).

However, less is known about how age and gender stereotypes operate specifically in digitally demanding recruitment contexts (Jensen et al., 2024). Digital roles place strong emphasis on adaptability, learning speed, and technological fluency. These attributes are often implicitly associated with youth and masculinity (Segkouli et al., 2023; Wynn and Correll, 2017). Research indicates that older workers are frequently perceived as less adaptable to technological change despite evidence of successful digital upskilling when training is provided (Kleissner and Jahn, 2020; Drazic and Schermuly, 2024). Yet most existing studies do not isolate demographic cues while holding qualifications constant. Consequently, it remains unclear whether digital transformation changes the *structure* of bias expression during recruitment.

A second unresolved issue concerns the level at which bias manifests. Digital recruitment systems are often described as mechanisms that reduce overt discrimination by standardizing evaluation procedures. Nonetheless, bias may shift rather than disappear. It may move from explicit hiring recommendations to competence-linked trait impressions and perceived skill assessments. Empirical research rarely compares these outcome domains within the same controlled design. As a result, we do not yet know whether demographic cues influence global hiring decisions, perceived digital skill gaps, or multidimensional trait evaluations differently.

Intersectionality further complicates this picture. Research on gendered ageism suggests that older women may experience compounded disadvantage in employment contexts (Handy and Davy, 2007; Mariano et al., 2020). Recent findings indicate that older women are less likely to receive access to training and development opportunities in technologically intensive environments (Oteng et al., 2024). However, intersectional effects may not be uniform across all evaluative dimensions. In digitally oriented roles, competence and vitality may become more salient than warmth-related traits, potentially producing differentiated rather than cumulative patterns of bias.

### Purpose of the study

The purpose of this study is to examine whether digital transformation restructures or intensifies age and gender bias in recruitment by altering which evaluative dimensions become most influential. To address this objective, the study employs a 2 × 2 between-subject factorial vignette experiment in which applicant qualifications are equivalent and only age and gender cues vary. This design allows for isolation of demographic cue effects on three analytically distinct outcomes: (a) hiring recommendation, (b) perceived skill gaps in digital and related domains, and (c) multidimensional trait impressions reflecting competence and integrity dimensions.

### Nature of the study

This study adopts a quantitative experimental design to isolate the causal effects of demographic cues in recruitment evaluation. A 2 × 2 between-subject factorial vignette experiment was selected because it allows manipulation of age and gender while holding qualifications constant. This design directly addresses the identified research problem by separating demographic inference from merit-based evaluation. Unlike survey-based or observational approaches, factorial experiments permit controlled testing of competing theoretical explanations regarding bias intensification versus bias restructuring. The design is appropriate for digitally demanding recruitment contexts because it enables examination of how demographic cues influence multiple outcome domains simultaneously, including hiring recommendation, perceived skill gaps, and multidimensional trait impressions. By distinguishing between explicit decisions and competence-linked evaluations, the study assesses whether demographic bias operates uniformly or selectively across evaluative dimensions.

### Research questions

The study is guided by an overarching research question “Does digital transformation intensify age and gender bias in recruitment, or does it restructure bias by shifting evaluative emphasis toward competence and vitality-related dimensions?” that is subsequently divided into three independent Research Questions:

*RQ1*: Do applicant age and gender influence HR professionals’ likelihood of recommending a candidate for a digitally demanding position when qualifications are equivalent?

*RQ2*: Do age and gender cues affect perceived skill gaps, particularly in domains associated with digital competence and adaptability?

*RQ3*: Do age and gender cues shape multidimensional trait impressions, especially those related to competence and vitality?

We argue that if digital transformation restructures rather than eliminates bias, demographic effects will be more pronounced in competence-related impressions and perceived digital skill domains than in explicit hiring recommendations. By testing these mechanisms within a controlled experimental design, the study advances understanding of stereotype expression under digital transformation and contributes experimental evidence to debates on ageism, gender bias, and intersectionality in recruitment.

### Rationale, originality, and significance of the study

Recent research confirms that age and gender disparities persist in hiring processes, including under conditions of increasing digitalization (Neumark et al., 2019; Lössbroek et al., 2021; Young et al., 2023). At the same time, digital transformation has redefined employability by emphasizing adaptability, technological fluency, and continuous learning. Although prior studies document discrimination, few isolate how demographic cues influence evaluations when digital competence is central and applicant qualifications are equivalent. Moreover, limited research compares explicit hiring recommendations with competence-linked trait impressions within a single controlled design. This gap constrains theoretical understanding of whether digitalization reduces bias, intensifies it, or restructures its expression. The present study addresses this limitation through a factorial experimental design that isolates demographic cue effects across multiple evaluative domains.

The study contributes original insight in three ways. First, it simultaneously manipulates age and gender while holding qualifications constant, allowing causal inference regarding demographic cue effects. Second, it examines bias across analytically distinct outcome domains—hiring recommendation, perceived digital skill gaps, and multidimensional trait impressions—thereby distinguishing between overt and competence-linked evaluative processes. Third, it situates these mechanisms within digitally demanding recruitment contexts, where competence and vitality are central evaluation criteria. By integrating stereotype theory with digital labor market transformation, the study extends existing research beyond general discrimination patterns to mechanism-specific analysis.

The study has implications for theory, practice, and policy. Theoretically, it clarifies whether demographic bias under digital transformation is cumulative or dimension-specific, contributing to debates on stereotype modernization and intersectionality. Practically, organizations increasingly rely on digital recruitment systems to address labor shortages in technology-oriented roles. If bias shifts toward competence-linked impressions rather than explicit hiring decisions, conventional diversity safeguards may fail to detect inequality. Identifying the level at which bias operates informs the design of structured evaluation tools, digital skill assessments, and bias mitigation strategies. At a policy level, understanding how demographic cues influence recruitment under digitalization supports evidence-based interventions aimed at improving labor market inclusion in aging and technologically evolving societies.

This study argues that in digitally demanding recruitment contexts, demographic bias is expressed less through explicit hiring recommendations and more through age-linked competence and vitality heuristics, indicating that digitalization restructures rather than simply intensifies stereotype expression.

## Methodology

### Research design

This study employed a quantitative 2 × 2 between-subjects factorial experimental vignette design to examine how applicant age and gender influence early-stage recruitment evaluations. Age (32 vs. 56 years) and gender (male vs. female) were manipulated through standardized résumés, producing four applicant profiles (F32, F56, M32, and M56). Each participant evaluated only one résumé condition. This structure isolates demographic cues while holding qualifications, experience, and résumé content constant. The design therefore allows causal assessment of how age and gender signals shape early screening judgments.

### Participants and data collection

The study included 608 HR managers from the Czech Republic who were actively involved in hiring decisions in their organizations. Participants were recruited through the European National Panels project administered by the research agency STEM/MARK. Data were collected using Computer-Assisted Web Interviewing (CAWI) between July and August 2021. Respondents were assigned to one of the four experimental résumé conditions through the survey platform, ensuring that each participant evaluated only a single applicant profile.

A quota sampling procedure ensured demographic representation by gender, age, education level, municipality size, and region. The final sample was balanced across key characteristics. Women represented 50.2% of respondents and men 49.8%. In terms of age distribution, 18.8% were under 35 years, 38.0% were between 35 and 50 years, and 43.3% were over 50 years. Educational backgrounds were also diverse. 10.4% of respondents had secondary education or less, 48.2% had completed non-university tertiary education, and 41.4% held university degrees. Participants represented a wide range of industries, which reduces the likelihood that the results reflect sector-specific hiring norms. Because the dataset was obtained from an external research provider, a separate institutional ethics approval was not required. The Human Research Ethics Committee at Mendel University in Brno reviewed the study protocol and confirmed that it complied with Czech legal and ethical standards. All participants provided informed consent, and responses were recorded anonymously to ensure confidentiality.

### Materials

To ensure ecological validity, participants reviewed a fictional job advertisement for the role of *Sales Representative for Retail Chains*. This job posting included realistic responsibilities such as client acquisition, customer service, negotiation, and coordination with marketing teams. The description was designed to reflect typical job expectations within the Czech labor market. Each participant evaluated one of several standardized résumés. The content of the résumés was held constant in terms of qualifications, professional experience, and formatting. Only two variables were manipulated: age (either 32 or 56 years) and gender (male or female). This design made it possible to isolate the effects of age and gender on evaluators’ perceptions while controlling merit-based differences.

### Procedure

After reading the job posting and assigned résumé, participants completed a structured online questionnaire. This process simulated a typical early-stage screening in recruitment and consisted of four components:

Recommendation Likelihood (Q1): Participants rated how likely they would be to recommend the candidate for the next stage of selection. A 7-point Likert scale was used to capture this evaluation.

Perceived Skill Difference (Q2): Respondents first rated the importance of 10 job-relevant skills for the position, including digital literacy, managerial competence, analytical thinking, and communication. They then evaluated the extent to which the candidate possessed these same skills. Ratings were recorded on a 0–4 scale, where 0 indicated “not required” or “not possessed at all,” and 4 indicated a “very high level.”

The perceived skill gap was calculated as the difference between the required level of each skill and the level evaluators believed the candidate possessed. Positive values therefore indicate that the candidate was perceived as lacking the required competence, whereas negative values indicate that the candidate was perceived as exceeding job expectations. This measure captures perceived person–job fit, reflecting how evaluators judge the alignment between candidate capabilities and role requirements during early recruitment screening.

Training Needs (Q3): Evaluators were asked whether they believed the candidate would require training upon employment. This measure added a practical dimension to the perceived skill assessment.

Trait Impressions (Q4): Participants rated the applicant on 30 semantic differential traits using 7-point bipolar scales. These traits were selected to capture evaluators’ impressions of the applicant’s personality, work style, and perceived emotional tone. These semantic items were later grouped into four broader conceptual categories based on prior literature: Instrumentality, Autonomy, Acceptability, and Integrity (Table 1).

**Table 1:** Semantic differential traits by conceptual dimension.

Instrumentality	Autonomy	Acceptability	Integrity
Progressive – Old-fashioned Thorough – Careless Wealthy – Poor Productive – Unproductive Busy – Idle Strong – Weak Healthy – Unhealthy Active – Passive Ordinary – Eccentric Exciting – Boring	Independent – Dependent Confident – Insecure Flexible – Inflexible Organized – Disorganized Liberal – Conservative Decisive – Indecisive	Generous – Selfish Attractive – Unattractive Cooperative – Uncooperative Friendly – Hostile Well-groomed – Ungroomed Trustworthy – Untrustworthy Tolerant – Intolerant Pleasant – Unpleasant Aggressive – Defensive	Optimistic – Pessimistic Satisfied – Dissatisfied Expectant – Resigned Hopeful – Discouraged Happy – Sad

### Construct operationalization and scale construction

The perceived skill difference score was operationalized as the difference between required skill level and perceived candidate skill level for each domain. This measure captures evaluators’ assessment of person–job fit, defined as the perceived alignment between candidate capabilities and job demands. In early-stage screening, recruitment decisions frequently rely on rapid judgments of fit rather than direct performance evidence. Stereotype-driven bias may therefore operate by lowering perceived competence relative to role requirements, producing a larger inferred skill gap even when qualifications are identical. By analyzing discrepancy scores rather than absolute ratings alone, the study captures evaluative mismatch as a mechanism of bias expression.

The 30 semantic differential traits were grouped into four conceptual dimensions: Instrumentality, Autonomy, Acceptability, and Integrity. Instrumentality and Autonomy represent competence- and agency-related constructs, reflecting effectiveness, adaptability, and decisiveness. Acceptability captures warmth- and communion-related attributes associated with interpersonal evaluation and social fit. Integrity reflects moral character and trustworthiness, which are central to hiring judgments but analytically distinct from competence. This structure aligns with established stereotype content frameworks that distinguish competence/agency from warmth/communion dimensions in social evaluation (Dovidio et al., 2010). Grouping items into theoretically grounded constructs enables examination of whether demographic cues selectively influence competence-linked impressions rather than uniformly affecting all evaluative domains. Internal consistency of the composite dimensions was assessed using Cronbach’s alpha. Reliability estimates indicated good internal consistency for the Instrumentality (*α* = 0.85), Autonomy (*α* = 0.83), and Acceptability (*α* = 0.87) scales, while the Integrity scale showed lower reliability (*α* = 0.45). These results support the aggregation of the semantic differential items into composite indices used in the subsequent analyses. All analyses were conducted in Python using the libraries Pandas, NumPy, SciPy, and StatsModels. Descriptive statistics were calculated for all variables across the four applicant profiles (F32, F56, M32, and M56). To match the factorial experimental design, two-way ANOVA models were estimated for each outcome variable, with age (32 vs. 56) and gender (male vs. female) specified as between-subject factors. These models tested the main effects of age, the main effects of gender, and their interaction. Where significant effects were detected, Tukey’s HSD post-hoc tests were conducted to examine pairwise differences between profiles.

Composite scores for the conceptual dimensions were calculated by averaging the items within each construct and the internal consistency of these scales was assessed using Cronbach’s alpha. Because multiple statistical tests were conducted across several outcomes, Holm-adjusted *p*-values were applied to post-hoc comparisons to reduce the risk of inflated Type I error. Effect sizes were reported as partial η^2^, and statistical significance was evaluated at *α* = 0.05.

## Results and discussion

Early-stage recruitment judgments are shaped by demographic cues even when competence information is limited. Experimental and field evidence shows that age and gender influence screening outcomes independent of objective qualifications (Neumark et al., 2019; Kricheli-Katz and Regev, 2021). Older applicants, and particularly older women, are often stereotyped as less adaptable and less digitally proficient. Such stereotypes shape perceptions of confidence, productivity, and sociability, which are central criteria in hiring decisions. According to the stereotype content model, competence and warmth structure these evaluations (Fiske et al., 2002). This study therefore examines how age and gender cues shape perceived skill fit and broader candidate impressions in early-stage recruitment.

**Figure 1:**
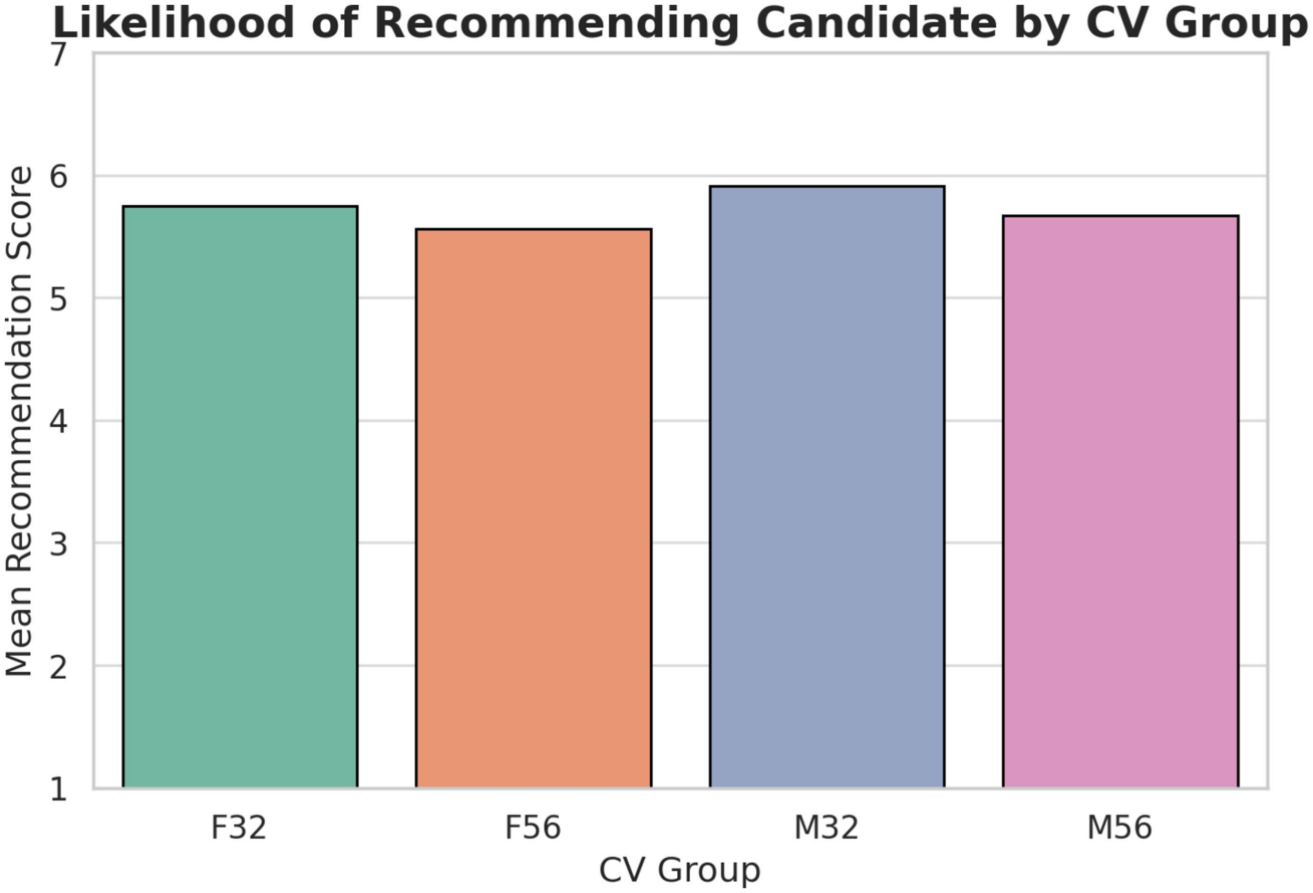
Mean scores for the likelihood of recommending candidates for the next round of selection, grouped by CV characteristics. Ratings were based on a 7-point Likert scale.

Explicit hiring recommendations did not show statistically significant demographic differences. Participants evaluated one résumé that varied only in age and gender, which isolated the effect of demographic cues. A one-way ANOVA revealed no significant difference in recommendation likelihood across profiles, *F*(3, 604) = 2.35, *p* = 0.079, *η*_p_^2^ = 0.004 (Figure 1). Tukey *post hoc* tests were also non-significant. However, descriptive patterns showed that younger candidates received slightly higher ratings than older candidates across both genders. This suggests that overt exclusion was not evident at the recommendation level, but a directional age preference was present.

The absence of statistical significance does not eliminate the possibility of subtle bias (Abrams et al., 2016; Dovidio et al., 2010). Younger applicants were consistently rated somewhat more favorably, which aligns with research linking youth to adaptability and digital readiness (Fasbender and Wang, 2017; Kricheli-Katz and Regev, 2021). At the same time, respondents may have been cautious in expressing direct preference due to social desirability concerns (Derous and Ryan, 2019). These dynamics indicate that explicit recommendation decisions may mask underlying evaluative tendencies. The findings therefore justify examining competence-linked and trait-level judgments to detect more implicit forms of demographic influence in early screening.

**Figure 2:**
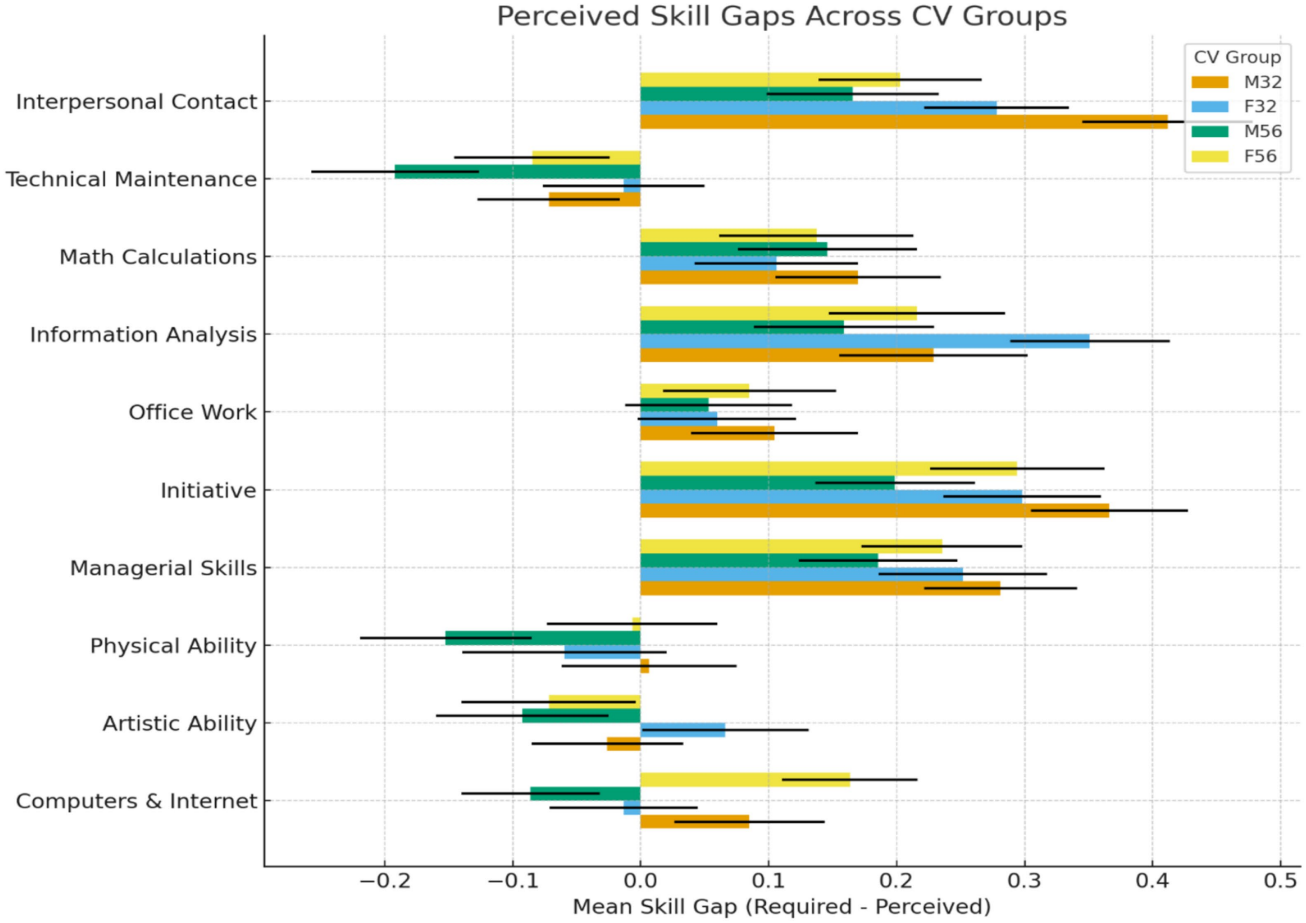
Perceived skill gaps across CV groups. Positive values indicate skills HR managers judged as lacking (required > perceived), negative values indicate perceived surpluses. Error bars show standard errors.

Perceived skill-gap evaluations revealed domain-specific demographic effects. Two-way ANOVAs were conducted across 10 competency areas, where higher values indicated a greater perceived gap between required and possessed skills (Figure 2). Most domains showed no significant main or interaction effects. However, specific competencies displayed significant age or age × gender patterns. This indicates that demographic cues did not produce a global deficit perception, but shaped judgments selectively in certain skill areas. These findings move the analysis beyond explicit recommendation and toward competence-based inferences embedded in skill evaluations (Figure 2).

Skill-gap effects were selective and concentrated in two domains (Table 2). Eight of the 10 competency areas showed no significant main effects of age or gender and no interaction effects. This indicates that evaluators did not uniformly associate demographic characteristics with lower competence. However, digital skills showed a significant age × gender interaction, *p* = 0.002 (Table 2). Tukey tests indicated that younger women (F32) were perceived as having significantly smaller skill gaps than F56, M32, and M56. Interpersonal contact also showed a significant age effect, *p* = 0.012, with younger women again receiving the most favorable ratings (Table 2). These results demonstrate that demographic influence emerged in role-relevant domains rather than across all competencies.

**Table 2:** Mean perceived skill gaps across 10 job-relevant domains, shown by candidate age and gender.

Skill domain	*p* (age)	*p* (gender)	*p* (Age × Gender)	Tukey *post hoc* summary
Digital skills	0.96	0.176	0.002	F32 < F56, M32, M56
Artistic/creative	0.116	0.384	0.582	None
Physical ability	0.454	0.573	0.134	None
Managerial skills	0.371	0.87	0.527	None
Independent work	0.177	0.829	0.198	None
Office work	0.84	0.92	0.553	None
Information analysis	0.137	0.194	0.635	None
Math/calculations	0.959	0.599	0.687	None
Technical maintenance	0.118	0.177	0.693	None
Interpersonal contact	0.012	0.449	0.181	F32 < F56, M32, M56

Skill gaps represent the difference between the level of skill required for the job and the level evaluators believed each candidate possessed, measured on a 0–4 scale.

The pattern reflects selective positive stereotyping rather than generalized disadvantage. Younger women were viewed as more digitally capable and more interpersonally effective than other groups, while the remaining profiles did not significantly differ from each other. This suggests alignment with contemporary ideal worker norms that emphasize adaptability, communication fluency, and digital readiness (Fasbender and Wang, 2017; Kricheli-Katz and Regev, 2021). The data do not show a universal penalty for older women. Instead, they indicate that evaluators favored candidates who fit current workforce prototypes. This domain-specific advantage clarifies that demographic bias in digital contexts may operate through selective expectancy rather than broad exclusion.

These findings refine stereotype-based models of recruitment. Traditional accounts of intersectional disadvantage argue that older women face compounded penalties due to assumptions of low competence and limited trainability (Neumark et al., 2019; Heilman and Parks-Stamm, 2007; Kenny et al., 2016). In contrast, the present results show that younger women received the most favorable evaluations in digital and interpersonal domains. This pattern aligns with the stereotype content model, which identifies competence and warmth as core dimensions of person perception (Fiske et al., 2002). Younger women were rated positively in domains that signal both digital proficiency and interpersonal effectiveness. This suggests that demographic cues can activate advantageous stereotype content when they align with role-relevant expectations.

The theoretical implication is that bias may operate through selective expectancy rather than uniform disadvantage. The findings show that stereotypes do not only penalize certain groups. They can also advantage groups whose attributed traits match current labor market norms. When digital fluency and adaptability are valued, candidates who fit this prototype receive favorable evaluations. This shifts bias from broad exclusion to targeted expectancy.

These selective advantages carry structural consequences. Favoring one subgroup in competence-linked domains raises the comparison standard for others. Even small differences at the screening stage can accumulate across later hiring, evaluation, and promotion decisions (Acker, 2000; Rhode, 2012). Over time, these patterns can reshape opportunity structures without visible discrimination. Bias in digital recruitment may therefore be subtle, domain-specific, and cumulative in effect (Trauth et al., 2016; Hirsch-Kreinsen, 2016).

**Figure 3:**
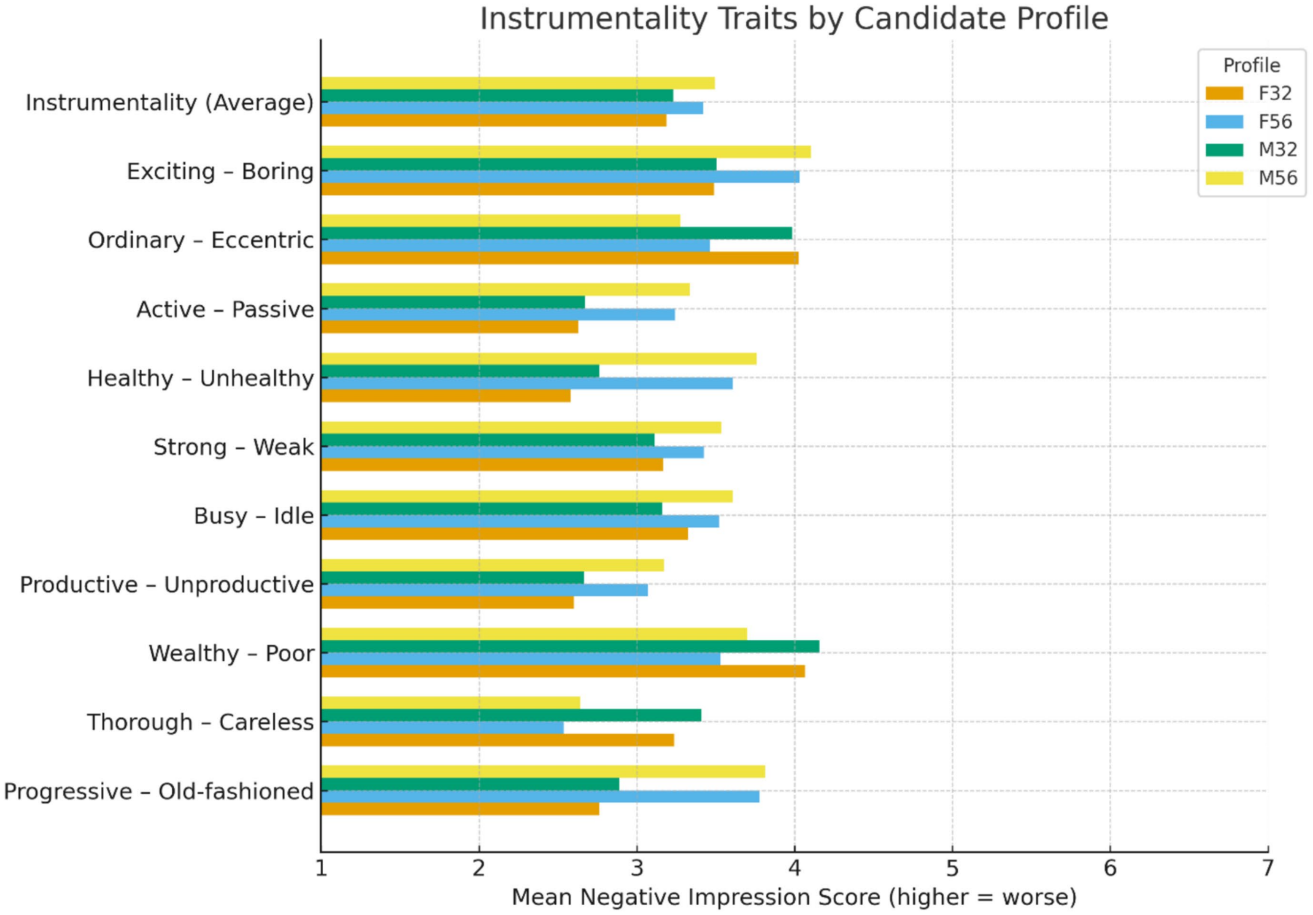
Mean scores for trait impressions across candidate CV groups. Trait impressions were rated on 7-point semantic differential scales.

Instrumentality evaluations showed a strong age-based competence penalty (Figure 3). Instrumentality reflected perceptions of competence, productivity, energy, and capability, with higher scores indicating more negative impressions. A two-way ANOVA revealed a significant main effect of age, *F*(1, 604) = 17.09, *p* < 0.001, *η*_p_^2^ = 0.028 (Figure 3). Older candidates were rated more negatively than younger candidates across all 10 traits. Gender showed no significant main effect, *F*(1, 604) = 0.99, *p* = 0.321, *η*_p_^2^ = 0.002 and the age × gender interaction was not significant, *F*(1, 604) = 0.07, *p* = 0.795, *η*_p_^2^ < 0.001. These results show that perceived capability declined with age independent of gender. Age cues therefore shaped core competence judgments at the earliest stage of screening.

*Post hoc* tests showed that the age penalty was strongest for older men. Tukey comparisons indicated that M56 received significantly more negative Instrumentality ratings than F32 and M32 on most traits, including productive, active, strong, healthy, and energetic, all *p* < 0.01. No significant differences emerged between F32 and M32 or between F56 and M56. Younger candidates were therefore evaluated similarly, while the sharpest decline in ratings was concentrated on the older male profile. This pattern indicates that the competence penalty was age-driven but expressed with greater intensity for older men.

These results show that age shapes core competence impressions at the screening stage. Instrumentality reflects judgments of capability, productivity, resilience, and vitality. Older applicants were rated as less capable despite identical qualifications. This aligns with evidence that age stereotypes include assumptions of lower performance and reduced adaptability (Ng and Feldman, 2012; Posthuma and Campion, 2009). These evaluations occurred before any behavioral evidence was available. Age cues alone were sufficient to reduce perceived competence at entry into the hiring funnel.

The stronger penalty for older men suggests a stereotype-role mismatch. Although gender showed no main effect, *post hoc* tests indicated that M56 received the most negative ratings. Older women were also rated less favorably than younger candidates, but the decline was less severe. This pattern is consistent with role congruity theory, which argues that men are expected to display strong agency and dominance, and violations of this expectation trigger harsher evaluation (Rudman et al., 2012). Age bias therefore interacts with gender norms in competence-linked domains.

These dynamics have structural implications for recruitment. Even when qualifications are controlled, early screening judgments relied on demographic heuristics. For roles that value energy, initiative, and drive, such competence-based filtering may remove older applicants before their skills are assessed. Over time, this process can reinforce structural age inequality in hiring systems.

**Figure 4:**
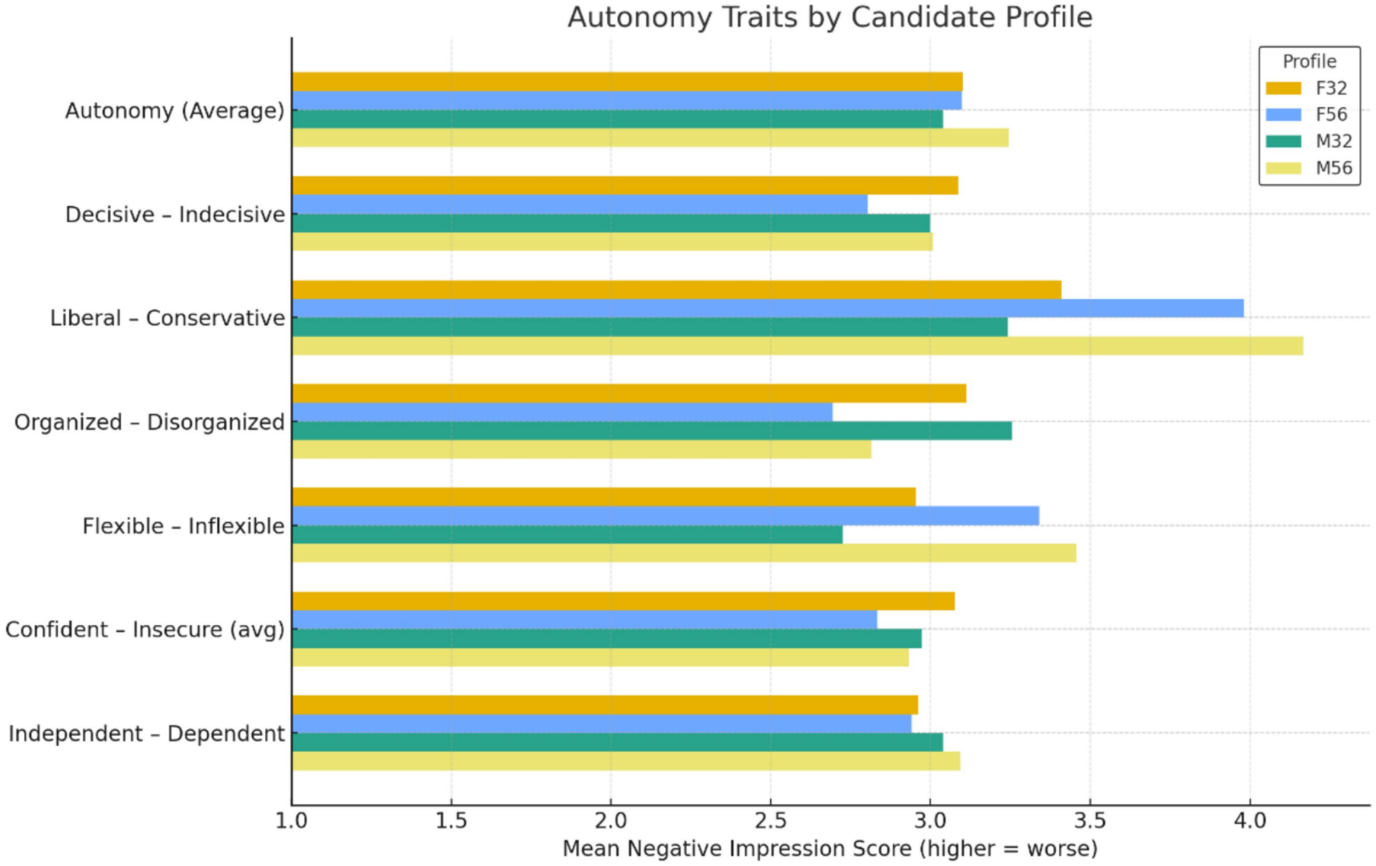
Mean negative impression scores on autonomy-related traits across candidate profiles. Higher scores indicate more negative evaluations.

Autonomy evaluations were not influenced by age or gender (Figure 4). Autonomy captured perceptions of independence, decisiveness, organization, and flexibility, with higher scores indicating more negative impressions. A two-way ANOVA showed no significant main effect of age, *F*(1, 604) = 1.92, *p* = 0.166, *η*_p_^2^ = 0.003 and no significant main effect of gender, *F*(1, 604) = 0.34, *p* = 0.561, *η*_p_^2^ = 0.001 (Figure 4). The age × gender interaction was also non-significant, *F*(1, 604) = 1.97, *p* = 0.161, *η*_p_^2^ = 0.003. Tukey *post hoc* tests confirmed that none of the four profiles differed significantly. These findings indicate that autonomy-related judgments remained stable across demographic categories at the screening stage.

Autonomy traits were insulated from demographic bias. Despite evidence that age stereotypes often include assumptions of declining capability (Ng and Feldman, 2012; Posthuma and Campion, 2009), no such pattern emerged for independence, decisiveness, or flexibility. Raters attributed autonomy consistently across age and gender profiles. These traits appear to function as professionally normative attributes rather than demographically coded signals.

This contrast highlights the selective nature of bias in screening. While instrumentality and vitality dimensions showed age penalties, autonomy did not. Bias therefore did not extend uniformly across all competence domains. This distinction shows that competence is multidimensional and that demographic effects may activate only in specific subdomains. Treating competence as a single construct would obscure these differences and weaken theoretical precision.

**Figure 5:**
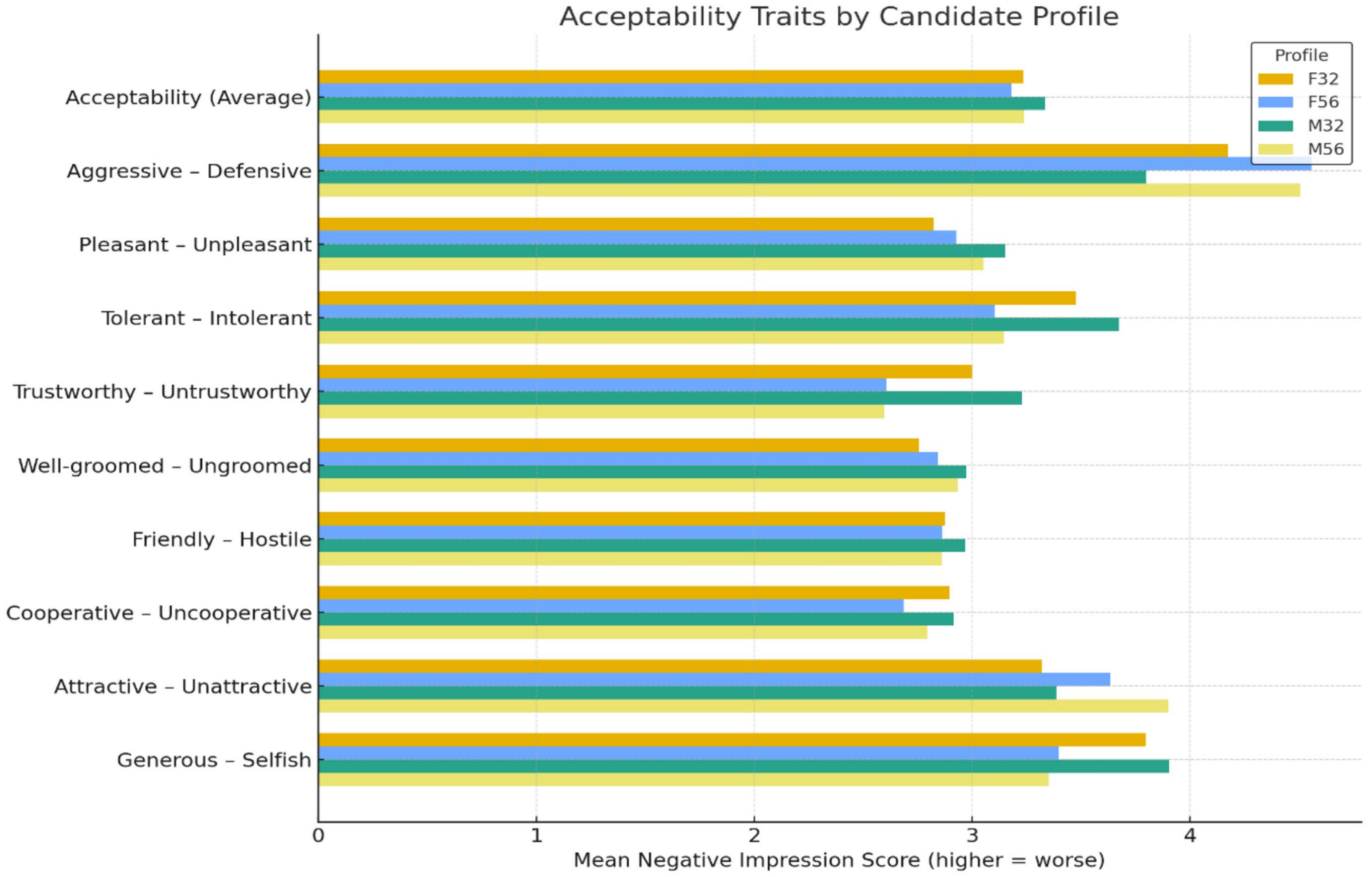
Mean negative impression scores on acceptability traits across candidate profiles. Higher scores indicate more negative evaluations.

Acceptability evaluations were not shaped by age or gender (Figure 5). Acceptability captured perceptions of interpersonal warmth and likability, with higher scores indicating more negative impressions. A two-way ANOVA showed no significant main effect of age, *F*(1, 604) = 1.44, *p* = 0.230, *η*_p_^2^ = 0.002 and no significant main effect of gender, *F*(1, 604) = 1.59, *p* = 0.208, *η*_p_^2^ = 0.003 (Figure 5). The age × gender interaction was also non-significant, *F*(1, 604) = 0.11, *p* = 0.740, *η*_p_^2^ < 0.001. Tukey *post hoc* tests confirmed that none of the four profiles differed significantly, all *p* > 0.39. These results indicate that interpersonal warmth judgments remained stable across demographic categories during early screening.

Warmth-related impressions were comparatively unaffected by demographic cues. When qualifications were held constant, age and gender did not shape perceptions of interpersonal likability. This contrasts with competence-linked stereotypes that often disadvantage older workers (Ng and Feldman, 2012; Posthuma and Campion, 2009). Evaluators attributed warmth similarly across profiles. Acceptability therefore appears to function as a baseline professional assumption rather than a demographic inference.

This stability reinforces the domain-specific nature of bias. Demographic heuristics influenced vitality and productivity dimensions, but not interpersonal warmth. Bias in early screening was therefore selective rather than global. Distinguishing competence-based from warmth-based judgments clarifies how unequal outcomes emerge within specific evaluative domains.

**Figure 6:**
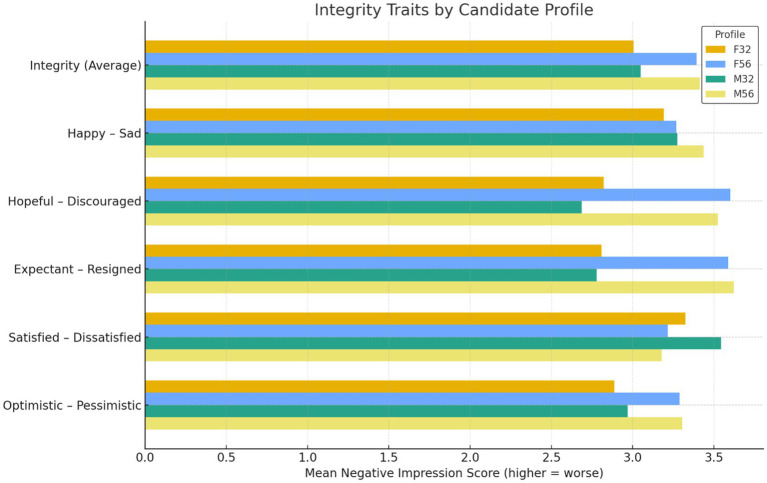
Mean negative impression scores on integrity traits across candidate profiles. Higher scores indicate more negative evaluations.

Integrity evaluations showed a strong age-based vitality penalty (Figure 6). Integrity reflected perceptions of emotional stability, optimism, and psychological resilience, with higher scores indicating more negative impressions. A two-way ANOVA revealed a significant main effect of age, *F*(1, 604) = 25.90, *p* < 0.001, *η*_p_^2^ = 0.041 (Figure 6). Older candidates were perceived as less emotionally resilient and less positive than younger candidates. Gender showed no significant main effect, *F*(1, 604) = 0.19, *p* = 0.663, *η*_p_^2^ < 0.001 and the age × gender interaction was not significant, *F*(1, 604) = 0.02, *p* = 0.881, *η*_p_^2^ < 0.001. *Post hoc* tests confirmed that younger profiles were rated more positively than older profiles, while no meaningful gender differences appeared within age groups. These results indicate that age, not gender, shaped emotional vitality judgments at the screening stage.

Age-linked stereotypes shaped perceptions of emotional resilience at the screening stage. Older candidates were viewed as less optimistic, less resilient, and less psychologically engaged despite identical qualifications. These impressions reflect common age stereotypes that frame older workers as less adaptable and less enthusiastic (Ng and Feldman, 2012; Posthuma and Campion, 2009). The judgments emerged before any behavioral evidence was available. Raters therefore relied on demographic cues rather than job-relevant information when forming vitality impressions.

Emotional-resilience bias was driven by age rather than gender. The absence of gender effects alongside a strong age effect shows that integrity judgments were anchored in age cues. Integrity traits functioned as a vitality heuristic. Candidates perceived as energetic and enthusiastic were evaluated more positively. Those perceived as emotionally fatigued were penalized.

This vitality heuristic has structural consequences. Emotional resilience is often framed as essential for demanding roles. When older applicants are assumed to lack this stamina, they may be screened out before their skills are assessed. Such early-stage filtering can reinforce structural age inequality in recruitment systems.

**Figure 7:**
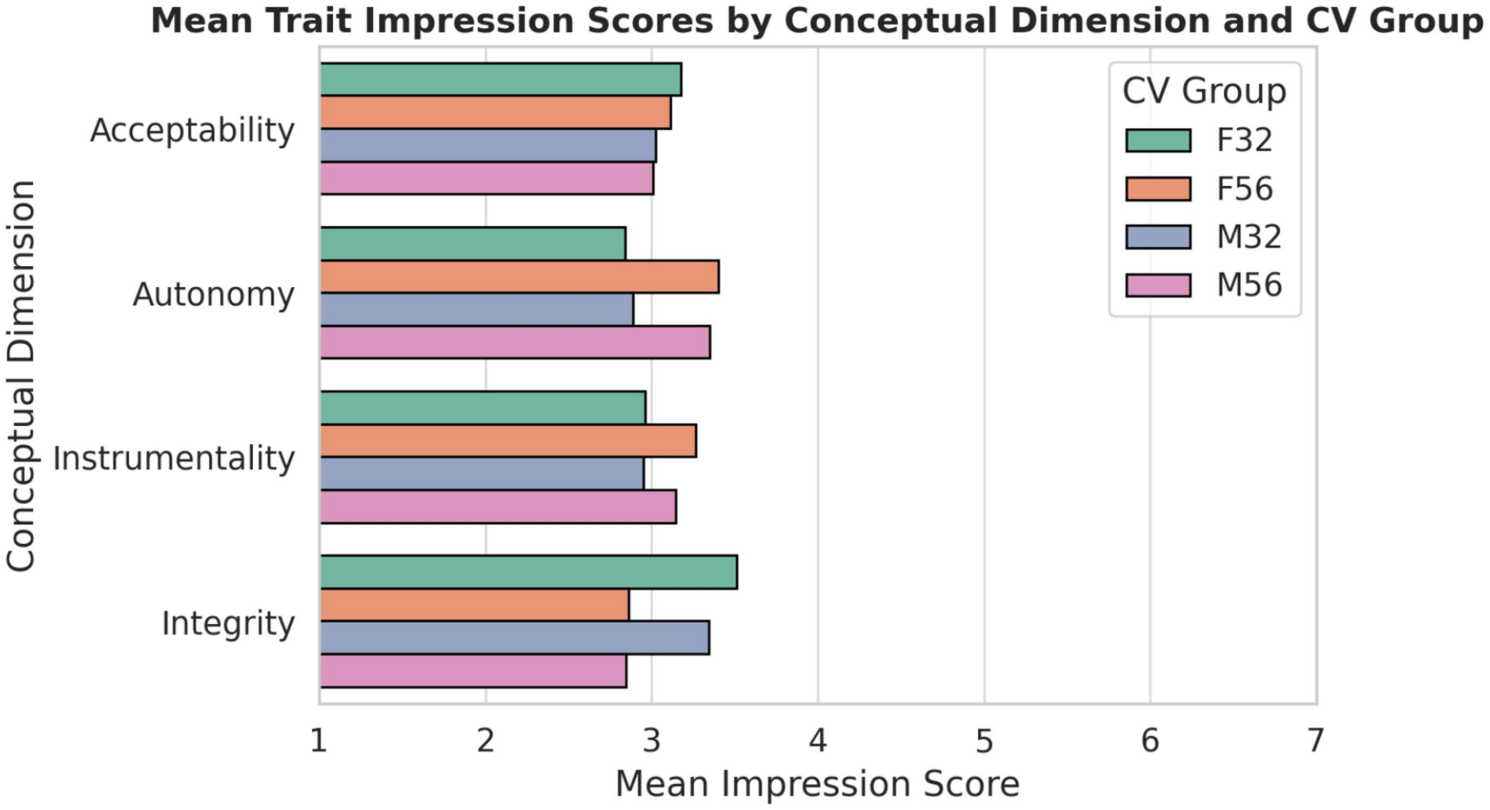
Mean trait impression scores across four conceptual dimensions—instrumentality, autonomy, acceptability, and integrity by candidate profile. Values are averaged across traits within each dimension using 7-point semantic differential scales, where higher scores reflect less favorable perceptions.

Across dimensions, age emerged as the primary driver of screening impressions (Figure 7). Older applicants were rated significantly lower than younger applicants on Instrumentality and Integrity, indicating perceived disadvantages in competence, vitality, and emotional resilience despite identical qualifications. In contrast, Autonomy and Acceptability evaluations did not differ significantly across demographic profiles, suggesting that demographic cues influenced competence- and vitality-related judgments more strongly than interpersonal warmth or independence. This pattern indicates that demographic bias in digitally oriented recruitment is dimension-specific rather than global, operating primarily through competence and vitality heuristics rather than interpersonal evaluation.

Bias in early hiring operated through competence judgments rather than interpersonal dislike. Older candidates were not penalized on warmth or likability, but on traits linked to capability and agency. This pattern aligns with research showing that age stereotypes frame older workers as less adaptable and less performance-driven (Ng and Feldman, 2012; Posthuma and Campion, 2009). The stronger penalty for older men is consistent with theories of gendered agency expectations (Rudman et al., 2012). These findings indicate that bias was subtle and competence-based, emerging before any evidence of skill was evaluated. Such early filtering can remove older applicants from the hiring pipeline prior to merit-based assessment.

The pattern of results suggests that demographic bias in digitally oriented recruitment is dimension-specific rather than global. Age influenced competence- and vitality-related impressions, as reflected in lower instrumentality and integrity ratings for older applicants, but did not affect autonomy or interpersonal acceptability. This asymmetry indicates that demographic cues primarily shape performance expectations rather than general social evaluations. In digitally demanding labor contexts, recruiters appear to rely on heuristic assumptions about energy, adaptability, and productivity when evaluating candidate capability. As a result, demographic bias operates less through overt exclusion and more through competence- and vitality-related inference processes during early screening.

Across outcomes, the findings converge on a coherent pattern: demographic cues did not significantly alter explicit hiring recommendations, yet they systematically shaped competence- and vitality-linked evaluations. Age effects were concentrated in domains associated with productivity, energy, digital fluency, and emotional resilience, while warmth-related dimensions such as acceptability remained comparatively stable across profiles. Skill-gap assessments revealed selective advantages for younger women in digital and interpersonal domains, whereas trait-level analyses showed consistent age penalties in instrumentality and integrity. Taken together, these results indicate that bias in digitally oriented recruitment does not operate primarily through overt exclusion, but through competence-based heuristics aligned with contemporary “ideal worker” norms emphasizing adaptability, technological readiness, and sustained performance. From the perspective of stereotype content theory (Fiske et al., 2002), this pattern suggests a contextual shift in salience: in digital labor contexts, competence-related dimensions become more decisive than warmth-related dimensions in shaping early evaluations. As a result, even small demographic differences in perceived vitality or digital readiness may accumulate across screening stages, subtly reshaping opportunity structures without manifesting as explicit rejection at the initial recommendation level.

### Interpreting the recommendation–impression discrepancy

A notable pattern in the findings is the absence of statistically significant differences in explicit hiring recommendation (Q1) alongside significant demographic effects in competence-linked trait evaluations and selected skill domains. This divergence suggests that demographic bias may operate at the level of evaluative inference rather than overt selection decisions.

One explanation concerns social desirability and evaluation apprehension. HR professionals are likely aware of legal and normative expectations regarding equal treatment, which may attenuate explicit discriminatory responses in global recommendation judgments (Stamarski and Son Hing, 2015; Derous and Ryan, 2019). In contrast, trait ratings and perceived skill gaps are framed as job-relevant assessments, making them appear more objective and professionally defensible. Under this account, demographic stereotypes may shape competence impressions without translating into explicit rejection at the recommendation stage. Future research incorporating social desirability scales or accountability manipulations could test this mechanism more directly.

A second explanation reflects the modernization of bias. Contemporary discrimination often manifests through subtle competence inferences rather than overt exclusion (North and Fiske, 2015). In digitally oriented roles, attributes such as adaptability, vitality, and technological fluency are central signals of employability. If these attributes are implicitly associated with youth, demographic cues may selectively influence competence-linked impressions while leaving global recommendations comparatively stable (Wynn and Correll, 2017). Mediation analyses examining whether competence impressions statistically transmit demographic effects to downstream decisions would help clarify this interpretation.

A third explanation involves decision-stage specificity. Early recommendation judgments may function as broad screening indicators, whereas trait impressions and perceived fit represent intermediate evaluations that shape later decisions cumulatively. Bias may therefore accumulate across stages rather than appear fully at the initial screening point (Lössbroek et al., 2021). A multi-stage experimental design tracking interview invitations and final selection outcomes would be required to evaluate this mechanism.

Taken together, the absence of overt differences in recommendation likelihood should not be interpreted as the absence of bias. Instead, the evidence indicates that demographic cues influence competence-linked evaluations in ways that may shape later selection outcomes. This pattern supports the study’s central claim that digital transformation may restructure the expression of bias toward competence and vitality dimensions rather than eliminate it.

### Significance of the study

Digital transformation is increasing demand for advanced digital skills while European labor markets face persistent skill shortages and demographic aging. Only 56% of individuals aged 16–74 in the EU possess at least basic digital skills (Eurostat, 2023), and more than half of enterprises attempting to recruit ICT specialists report difficulty filling vacancies, with rates exceeding 70% in Czechia (Eurostat, 2023). At the same time, the proportion of workers aged 55 and older continues to rise across Europe (OECD, 2022). In this context, biased early-stage screening can generate structural inefficiencies by narrowing applicant pools and excluding qualified candidates from digitally demanding roles.

The findings are directly relevant to HR leaders, policymakers, and workforce development institutions. They demonstrate that demographic cues shape competence- and vitality-linked impressions even when explicit hiring recommendations remain stable. This suggests that recruitment systems may reproduce inequality through subtle competence inferences rather than overt exclusion. Structured screening tools, work-sample assessments, and stage-specific audits of digital skill evaluation can therefore reduce reliance on demographic heuristics.

The study contributes to the literature by providing controlled experimental evidence from a Central European context and by distinguishing between explicit recommendation and competence-based evaluation mechanisms. It advances theory by showing that, under digital transformation, competence-related stereotype dimensions become more salient than warmth-related ones in early hiring judgments. This distinction clarifies how bias may be restructured rather than eliminated in digitally mediated recruitment.

### Recommendations for practice

Because bias in this study concentrated in competence- and vitality-linked judgments, interventions should target early screening structures that invite such inferences. Organizations should implement a structured résumé screening rubric with predefined weights for each job-relevant competency. Evaluators must complete competency scoring before entering any overall recommendation, and recommendation fields should remain inaccessible until all criteria are scored. Age-salient cues such as date of birth and graduation year should be masked during the initial screening stage wherever legally and technically feasible to reduce activation of vitality-based assumptions prior to competence evaluation.

Digitally demanding roles should incorporate standardized digital task assessments prior to résumé-based elimination so that advancement decisions rely on demonstrated performance rather than inferred technological readiness. Rejection decisions should require written justification linked explicitly to rubric criteria, and vague fit-based explanations should not be accepted. Organizations should conduct stage-by-stage progression audits by age and gender to identify disparities at screening, interview, and selection stages. Evaluator training should address vitality-based stereotyping directly, including structured exercises that demonstrate how assumptions about adaptability, energy, and digital fluency may be unconsciously linked to age. Together, these measures shift early recruitment decisions from inferred competence to verified capability and reduce the accumulation of demographic bias across hiring stages.

## Conclusion

This study set out to examine whether digital transformation reduces demographic bias in recruitment or restructures the way it is expressed. The findings indicate that bias under digitalization operates less through overt rejection and more through competence- and vitality-linked inferences. Explicit hiring recommendations did not differ significantly across demographic profiles, yet age cues systematically shaped perceptions of productivity, energy, digital fluency, and emotional resilience. Warmth-related dimensions remained comparatively stable. These results suggest that, in digitally oriented labor contexts, competence-related stereotype dimensions become more salient than warmth-related ones. Digitalization does not eliminate bias; it shifts it toward judgments of adaptability and technological readiness aligned with contemporary “ideal worker” norms. At the same time, the study does not demonstrate that these competence-linked impressions inevitably translate into final hiring exclusion. The design captures early-stage screening judgments rather than multi-stage recruitment outcomes. This limitation matters because bias may accumulate gradually rather than appear in a single decision point. The absence of significant differences in recommendation likelihood should therefore not be interpreted as evidence of fairness, but as an indication that modern bias may operate through evaluative pathways that are less visible yet potentially consequential over time.

The contribution of this study lies in clarifying how demographic cues function under digital transformation: not as uniform disadvantage mechanisms, but as selective competence heuristics that reshape early evaluation processes. By distinguishing between explicit recommendation and competence-based impression formation, the study advances theoretical understanding of stereotype content in digital labor markets and provides an empirical foundation for redesigning recruitment systems. Recruitment processes that prioritize demonstrated capability over inferred vitality can reduce the accumulation of stereotype-driven competence bias and improve both equity and talent utilization in digitally transforming economies. Digital transformation may therefore not eliminate demographic bias in recruitment but instead restructure it toward competence- and vitality-related inference processes during early screening.

### Research limitations

This study has limitations that constrain interpretation and generalization. The experimental design used simulated CVs in a controlled setting. This approach isolates demographic cues but does not capture the full complexity of real hiring decisions. Factors such as organizational culture, time pressure, and stakeholder influence were not present. Screening behavior in practice may therefore differ from observed patterns.

The sample context also limits generalizability. Participants were HR managers from the Czech labor market. This context provides valuable insider insight but reflects specific demographic and institutional conditions. Hiring norms and diverse frameworks vary across countries. The findings should therefore be interpreted as context-sensitive rather than universally generalizable.

The scope of measured constructs was limited. The study focused on four competence-related dimensions and did not examine attributes such as teamwork, motivation, leadership potential, or contextual fit. Hiring decisions in practice reflect a broader mix of explicit criteria and implicit expectations. The current design therefore captures only part of the evaluative process.

Future research should expand construct and context coverage. Studies should include a wider range of competencies, diverse job roles, and multiple sectors. Field data or real applicant pools would strengthen external validity. Such designs would clarify how demographic cues interact with demonstrated skills in actual selection processes.

### Directions for future research

The impact of digital transformation on hiring, particularly how AI tools influence age and gender biases, requires further investigation. While automation can reduce subjective hiring elements, poorly designed systems may perpetuate existing biases. Future studies should focus on strategies to ensure digital hiring processes are equitable and competency-based rather than demographic-driven. Research should also expand to explore competencies like adaptability, creativity, and emotional intelligence, which are increasingly valued in the workforce. Understanding how age and gender biases affect these competencies will provide a fuller picture of hiring disparities. Industry-specific bias patterns warrant exploration, as sectors like technology may exhibit different biases compared to industries such as healthcare. Tailored interventions for each sector could address unique cultural dynamics and hiring practices.

Additionally, research into the psychological mechanisms behind biases in hiring decisions could lead to more effective interventions to counteract unconscious biases. This would inform training programs aimed at promoting fairer evaluations. Finally, examining the economic impact of bias-free hiring practices could help organizations understand the tangible benefits of equitable recruitment, including improved productivity, satisfaction, and retention.

## Data Availability

The raw data supporting the conclusions of this article will be made available by the authors, without undue reservation.
